# Lived Experiences of Learning to Use a Long Cane: The Importance of Integrating Perceptual, Existential, and Social Dimensions for Active Cane Use

**DOI:** 10.1177/10497323241277111

**Published:** 2024-10-10

**Authors:** Inger C. Berndtsson

**Affiliations:** 1 Department of Education and Special Education, 3570 University of Gothenburg, Gothenburg, Sweden; 2 Stias Stellenbosch Institute for Advanced Study, Stellenbosch, South Africa

**Keywords:** learning experience, visual disability rehabilitation, lifeworld phenomenology, orientation and mobility, long cane

## Abstract

How do people who are blind or visually impaired experience learning to use a long cane? This question is of paramount importance for planning and delivering rehabilitation programs and orientation and mobility (O&M) training. Until now, research into learning to use a long cane has focused primarily on technical and professional aspects, paying little attention to the lived experience of the learning activities that are offered in the field of O&M. This extensive qualitative study adopts a lifeworld phenomenological approach and sets out to examine the pedagogical processes within rehabilitation, focusing on the learning experiences of people with impaired vision. The methods used included participant observation during O&M training sessions and recurrent narrative interviews with three research subjects. The results show that learning to use the long cane has perceptual, existential, and social dimensions which are intertwined processes that relate to mind and body, body–world relations, and human existence and society. Learning to use a long cane has in this study been interpreted as embedded with cultural meaning about disability. Further, the habitual use of the cane promotes adaptation to visual impairment but also to build new body–world relations. The lifeworld theory and its methodology have contributed to theoretical evidence and rigor throughout. The results bring new interpretations to the field of O&M and are a relevant basis and valuable for pedagogical rehabilitation as it highlights the importance of taking the individual’s lifeworld and needs into consideration when teaching someone how to use a long cane.

## Introduction

This article sheds light on the lived experiences of people who, due to visual impairment or blindness, need to learn to use a long cane for orientation and mobility (O&M) and on pedagogical processes when learning to use this assistive device. The existing knowledge base and practice in this field generally emphasize technical issues of the long cane (Hersh, 2015), tending sometimes to objectify the person rather than viewing them as a learning subject. Research into people’s own learning experiences in O&M is rare. In accordance with the importance of psychosocial aspects related to long cane use, there is surprisingly little research on these aspects (Welsh, 2010), indicating a research gap. In a study by Penrod et al. (2023), the psychosocial aspects are even ranked as the second most important knowledge area for O&M instructors, which also points to its importance. There is a need for deepened knowledge about how people feel and experience starting to use a long cane and how the cane mediates between emotional aspects and identity. This knowledge is needed to organize teaching and training in the best possible way. The lifeworld phenomenological approach offers a basis for empirical research which takes into consideration the lives of human beings, living in a social world (Bengtsson, 2013b). Starting from the lived experiences of individuals and their lifeworld enables integration of both bodily and social aspects of O&M, aspects that are all relevant for studying pedagogical processes within rehabilitation.

A lifeworld phenomenological perspective involves challenging and questioning conventional understandings of O&M, meaning the field of knowledge and professional discipline that teaches people with visual impairment and blindness the skills to find their way independently and walk safely (Wiener et al., 2010). One common definition of orientation and mobility is as follows:

*Orientation* refers to knowledge of one’s distance and direction relative to things observed or remembered in the surroundings and keeping track of these spatial relationships as they change during locomotion; *mobility* is the term used to describe the act of moving through space in a safe and efficient manner. (p. xv)

Since the development of O&M techniques in the United States after World War II, the primary focus has often been on the individual’s grasp of long cane techniques and various technical issues relating to other technical aids and environmental adaptations (Wiener et al., 2010) or wayfinding tools (Parker et al., 2021). More concretely, the long white cane is moved back and forth in front of you, for detecting obstacles, when moving forward. When Sauerburger and Bourquin (2010) discuss teaching the use of a long cane step by step, they present strategies to ensure that people learn the different steps with the cane properly from a manual performance of skills to notice and use information provided by the cane. This raises questions of a more phenomenological nature about how the learning of the cane can be understood when it becomes part of people’s concrete relating to the world. In his book about the science and teaching of O&M, Jacobson (2013) talks about a holistic approach, but it is not quite obvious what that means. When it comes to learning, the focus has traditionally been on behaviorism and cognitive theories (Jacobson & Bradley, 2010). However, human behavior and cognition alone cannot explain how O&M skills are actually learned and used. The phenomenon focused on in this study is how people with visual impairment experience learning to use a long cane in their everyday life. What feelings are aroused in people who need to use a long cane, how should one understand a person’s relationship to the world when using it, and not least, what does it mean for learning that the cane is white and that the person thereby becomes associated with often stereotypical images of blindness? All in all, a complexity emerges here when someone is learning to use a long cane, where pedagogical processes are studied from individual and shared experience.

### Experiences of Learning and Using a White Cane

Using a lifeworld approach means to view the individual in relation to his or her world and other people. Intersubjectivity is thus a central aspect of the lifeworld. For persons with visual impairment or blindness, research has shown lived experiences of exposure to social stigmatization (Goffman, 1972), whereby a blind person’s experience is being reduced to his or her disability (Karlsson, 1999). This is even more exposed by using a white cane, as shown by Berndtsson (2001) in a lifeworld phenomenological study where people with visual impairment or blindness were interviewed. This shows that learning to use a long cane is also fundamentally bound up with how the individual interacts with other people and with their environment (Berndtsson, 2001; Hammer, 2016). The white cane is the external visible sign that the body has changed. Once you start using it, you cannot escape anymore, and this is where experiences of otherness emerge in people (Berndtsson, 2001). This phenomenon has also been understood in terms of experiencing oneself as stigmatized, where the ideas that people with visual impairment encounter about themselves are internalized in the white cane (ibid.) which can also result in immobilizing a person (Wainapel, 1989). As both Bäckman (2023) and Berndtsson (2018) have elaborated, the long cane is therefore not a neutral tool; it contributes to a changed identity. The visibility of the long cane “mediates the user’s value, and blind people are often assigned a lower value than so-called ordinary people” (Berndtsson, 2018, p. 146). This is also accentuated by Kudlick (2005), who writes about “public responses to the white cane as the ultimate symbol of helplessness and powerlessness” (p. 1590). This means that people are reluctant to appear in public with their white cane. This experienced intersubjective aspect negatively affects the ability to independently carry out O&M. Many people with blindness also find it difficult to start using a cane due to psychological aspects or stigma (Hersh, 2015). A survey has also found that there were high reported rates of underuse of mobility devices, including long canes, which was hypothesized as being related just to stigma and the adaptation process (Brunes et al., 2024). Often, there is an ambivalence in relation to the white cane from the users’ point of view, which relates to the tension between normality and deviation, where the white cane prevents the person to pass as a “normal” and able-bodied person (Bäckman, 2024). Worth (2013) emphasizes that one strategy young people with visual impairment used was to conceal their visual impairment, in order to “pass”; but also, being a “competent spatial actor” challenged other people’s expectations about being a blind person. On the other hand, dos Santos et al. (2022) have found that having a close relationship with disabled people will have a positive impact on the perception of the long cane as a symbol of disability.

From a theoretical phenomenological perspective, Merleau-Ponty (2012) has elaborated on the long cane as an extension of a person’s own body. However, in empirical studies, Berndtsson (2001, 2018) has found similar statements regarding embodiment from persons with blindness in relation to learning to use a long cane. Embodiment as a phenomenon has also been found related to more physical impairments and assistive technology where both Standal (2011) and Papadimitriou (2008) go into the issue of re-embodiment when learning wheelchair skills. Standal argues that this process affects the wheelchair user’s being-in-the-world and expresses the close relationship between acquiring new habits and incorporating the wheelchair into the person’s own body. Papadimitriou discusses how becoming “en-wheeled,” learning to use the wheelchair and making it part of daily life, includes processes in which the wheelchair becomes an extension of the self, even though it also functions as a symbol of incapacity. Another study using a lifeworld approach is by Pettersson et al. (2005), with a focus on how assistive devices are experienced by the spouses of people who have had a stroke. It was found that the lived experience of assistive technology could be described by lifeworld existentials, described by van Manen (1997) as lived body, lived space, lived time, and lived human relationships.

The long cane has also been interpreted as a tool for conquering the experienced new lifeworld related to visual impairment and blindness and how it brings forth interpretations of how persons after becoming blind could build and conquer the new lifeworld by using the long cane (Berndtsson, 2018). This idea was introduced in an earlier study where the cane in its habitual use was seen as integrated into the lived body and part of the acquisition of a *world* (Berndtsson, 2001). Bouman (1962) has also taken a phenomenological approach in his work, insisting on exposing his theoretical ideas to the perspectives of people who were, or were becoming, blind. Among other things, he draws attention to how extremely difficult it is to transition from the sighted world to the blind world, as he puts it.

To summarize, there are empirical studies that describe the use of the cane from an intersubjective perspective with a focus on perceived stigmatization, there are studies describing the embodied aspects when starting to use a long cane, and finally, the cane is highlighted as a tool for conquering a new world. It is apparent, then, that there is limited knowledge of how individuals who become visually impaired or blind experience learning O&M skills. The only identified studies in this respect are autoethnographic (Bäckman, 2020; Kudlick, 2005) and qualitative self-studies (Fourie, 2007).

The aim of this study is to investigate how learning to use the long cane can be understood from a lifeworld phenomenological perspective, thus putting lived embodied experience and lifeworld theory into focus. The following questions will be addressed in depth: (1) How does the process of learning to use the long cane start out from individuals’ lifeworld experience, and which dimensions of that experience stand out as especially important? (2) How can the lived experience of learning orientation and mobility skills be interpreted through the frame of lifeworld ontology and theory?^1^

## Lifeworld Theory: Intertwinement of Body and World

The theory of the lifeworld draws on phenomenology (Husserl, 1973) and, as developed by Bengtsson (2005, 2013b), takes a pluralistic and integrative view of reality. In contrast to traditional ways of understanding reality, the lifeworld offers an integrative view of life and the world around us (ibid.). The world is always seen from the point of view of the individual’s own life while, at the same time, the world always affects that life. This means that our life and the world around us intermingle with each other and are not in fact separate entities. When there is disharmony in the world around us, the body changes, and this in turn changes the individual’s world. The principal characteristic of a lifeworld is thus “both, and” rather than “either, or” (Bengtsson, 2013b, p. 6). From this view, each person has his or her own lifeworld, which is experienced through their existence, expressed as “being-in-the-world” (Heidegger, 2013). It is through our human bodies or *lived bodies* that we experience the world around us (Merleau-Ponty, 2012). According to this theory, the body and the mind are closely intertwined. However, the lifeworld is also a social entity (Schutz, 1962), as our respective lifeworlds meet and intermingle with each other. For this analysis, it is obvious that experienced blindness radically reshapes body–world relations (Berndtsson, 2001, 2018). At the core of the lifeworld are the activities we perform every day. When we commit ourselves to certain activities and projects, we engage with the world. It is this daily round of routine activities which very often typifies our everyday life. However, this “habitual body,” as described by Merleau-Ponty (2012), is often affected by a disability of some kind.

The long cane is in this respect not an object per se but instead “seen *as something* and call for action on our part” (Berndtsson et al., 2007, p. 263), and should be seen in its’ “utility quality” and possibility to be used (Bengtsson, 2013b, p. 5). Where the body–world relations are broken, the long cane “takes shape in the space between life and world, where its use contributes to the development and refinement of relations to the world” (Berndtsson, 2018, p. 150). However, how this is done over time, and how the cane contributes to restored body–world relations within the context of rehabilitation, is an empirical question relevant for this study. Following this line of reasoning, learning is seen as the activity that again merges body and world, in a kind of restored or new lifeworld for that individual (Bengtsson & Berndtsson, 2015). The variability of the lifeworld with its continuing shifting horizons (ibid.; Berndtsson, 2001; van Peursen, 1977) is a prerequisite of learning to take place. Also, the human being as embodied, integrating body and soul in a bodysubject (Merleau-Ponty, 2012) points to the individual as an embodied actor when engaged in learning, both related to the regional lifeworld at a rehabilitation center and in everyday life and society (Bengtsson & Berndtsson, 2015; Schutz, 1962).

## Design and Method

The lifeworld approach is characterized by its open and flexible methods and based on the lifeworld, both as ontology and lived reality (Bengtsson, 2013b). The methods selected for this study adhere to the qualitative tradition and include a combination of participant observations (Hammersley & Atkinson, 2019; Taylor et al., 2016) and qualitative open-ended interviews (Kvale & Brinkmann, 2009). As O&M is a physical activity, it seemed very appropriate to make use of participation in lessons as a way to study the participant’s bodily and perceptual way of being-in-the-world, and a narrative approach was adopted primarily because of this study’s focus on feelings, reflections, and lived experience during O&M training, “the aim being to recover and strengthen the voice of the lifeworld” (Mishler, 1991, pp. 142–143), striving for rich descriptions of the lived phenomenon being studied (Finlay, 2009). Taken together, these two methods gave the researcher the possibility to further reflect on and together discuss and talk about the meaning of the observed O&M lessons together with the participants. All of them were also invited to write an audio diary to document their own learning process, but only one person (Alice) used this possibility. Throughout the process, the research was carried out with great care and respect for the people who participated in the study and their perspective, trying to establish a we-relation (Berndtsson, 2016; Schutz, 1967). For example, the choice of location for the interviews was based on participants’ preferences. The researcher also consistently tried to be sensitive and respectful of what was said during the interviews.

### Participants

Three people (one woman and two men) who were due to participate in individual O&M training with a long cane were purposely selected for this study.^2^ Selection took place just as they were beginning a course of active rehabilitation and training at a rehabilitation center. They are aged between 31 and 61 and are each in need of a long cane due to facing difficulties with their orientation and mobility. Alice is visually impaired and Peter is blind: in both cases, they acquired these disabilities in adulthood. Dick has been visually impaired since his teens. Both Alice and Dick have restricted fields of vision and lack twilight vision. The sample represents a variety of ages, visual impairment, and gender, but also a variety of housing, family circumstances, and work situation, allowing insight into different lifeworlds.

### Participant Observation: Sharing the Participants’ Lifeworld

With the permission of the rehabilitation center and its employees, the researcher participated in and observed the O&M training activities, being there to share the world and try to come close to how the world is experienced by the participants (Beekman, 1984; see also Friberg & Öhlén, 2010), as a way of walking alongside the persons (Beck et al., 2024). These were not specifically organized around their participation in the study; rather, the researcher was present during the normal, planned activities for each person. Observing people’s use of tools and ongoing activities is a research tool to gain knowledge about other’s lifeworlds (Berndtsson, 2016). In total, 31 O&M training sessions were observed, each session lasting from 2 to 3 hours. Eight of these sessions were with Alice, 9 with Dick, and 14 with Peter. The sessions took place both indoors and outdoors, and both in the participants’ local environments and in the city center. The bulk of the observed sessions comprised basic skills training with the long cane indoors in a room and in corridors as well as outdoors in park areas, in urban environments and using public transport (Alice and Dick), and for Peter orientation activities in his neighborhood as well as the use of public transport (Peter). All sessions were supervised by the same O&M trainer. Her strategy was to develop a good relationship with the participants, laughing and letting them feel comfortable in the training situations. She basically followed the established guidance for O&M training (Jacobson, 2013). The lifeworld approach gives meaning to participant observations of lived bodies in O&M learning activities as “mental life is expressed in the body, and bodily movements are mental” (Bengtsson, 2013b, p. 8). During observations, the researcher tried to avoid interrupting the lessons as far as possible, refraining from interaction other than at the beginning and end of each session, except when drawn into the session by the trainer or participants themselves. Sessions focused on building the relationship between the participant’s lived body and the environment by using hearing, echolocation, touch, and smell and on developing their ability to make use of information transmitted by the long cane. Participants’ reactions and dialogues with the trainer were also observed. Exhaustive notes were taken during observation and afterward written up on a computer (Hammersley & Atkinson, 2019).

### Interviews: Narrative and Reflection

The material for this article is based on (1) a life-story interview (Eastmond, 1996) focusing on how their lives had changed because of visual impairment; (2) interviews about learning O&M skills and how participants were making practical use of their new knowledge in their everyday lives; and (3) follow-up interviews within the year.

The number of topics discussed differed from one interview to another. Four fairly lengthy interviews were carried out with Alice, and three with Peter, in their homes. Dick was interviewed on eight occasions at the health agency in close connection to his O&M training. The number of interviews varied depending on where they were conducted and the length of the journey. All interviews were taped, and the collected material per participant comprised 11 hours with Alice, 11 hours with Dick, and 6 hours with Peter.

During the interviews, the researcher showed great interest in the participants’ experiences and what they had to say, in order to take part of their embodied experience (Bengtsson, 2013a). The relaxed context of these interviews, which in practice were more like dialogues, inspired participants’ confidence. At the beginning of each interview, the interviewer asked for details of any significant learning experiences since the previous interview. The interviews were based on interview guides with open questions, but the interviewer, while aware of the guidelines, focused primarily on what she was being told by the interviewee and tried to create an atmosphere that would encourage them to relate personal anecdotes and narratives, as these are often very telling and can yield excellent spontaneous examples of lived experience (Mishler, 1991). The interviews were also a good opportunity to exchange thoughts on the topics and observations from the lessons in a friendly, chatty atmosphere. The interviewer drew on her own experiences and notes from the participant observations as a basis for discussion and reflection during the interviews. This implies the researcher’s embodied existence in the practical world of research (Bengtsson, 2013a). All in all, the interviews created ideal opportunities to talk about and investigate the lived embodied experience of those undergoing O&M training and practice, thereby interweaving observations and interviews. Despite the variations, it is judged that all three participants have provided rich and informative interview accounts that help to answer the research questions. All interviews were transcribed verbatim by the researcher.

### Interpretation and Lifeworld Hermeneutics

The resulting material has been analyzed in great detail several times over, a process that in practice already commenced during the interviews, participant observations, and process of transcription (Bengtsson, 2013b; Ödman, 2007). To begin with, an overview was developed of each person’s life changes, based on the life-story interviews. It was vital to understand how the participants had formerly lived their lives and what had changed. Next, a thematic analysis was performed for each participant, identifying the central themes in their O&M training and learning experience, based primarily on the interviews and, to a lesser extent, the observation notes. Each participant’s own interpretation of their lived experience was articulated in these analyses. Finally, the researcher used hermeneutical interpretation (Heidegger, 2013; Ödman, 2007) to attempt to understand the meaning to participants of learning O&M skills, drawing on the observations, the narrative material gathered, and the audio diary. Tentative interpretations were carefully formulated and tried out. The hermeneutic processes were guided by identifying various parts and the whole of an intermittent and interactive process, as in a hermeneutic circle or spiral, where both parts and the whole were constantly formulated and reformulated until the best and most relevant interpretations can be said to have achieved credibility (Ödman, 2007). The resultant interpretations in this case are those which seemed to fit best the overall body of data. In this last phase, lifeworld theory was a useful tool with which to enhance the understanding of lived experiences (Bengtsson, 2013b; Berndtsson et al., 2007) integrated into the interpretations through abduction (Berndtsson & Vikner Stafberg, 2022).

### Ethical Considerations

An ethical approach has been applied throughout the study. First, a meeting was organized with each participant where they were verbally informed of the ethical considerations, that participation is voluntary and what it entails, that they can withdraw from the study at any time, but also that identifiable data will be removed from the material and publications. They were also informed of the possibility of accessing texts that concern them before they are published. The information was also communicated in a medium they could read, often by email. Having decided to join the study, they signed the consent form. All names in the text have been replaced by pseudonyms. The research has been approved in its current form by a national research ethics committee.

## Results: Interpretations of Learning to Use the Long Cane

For all of the participants, visual impairment or blindness has been interpreted as a break in their lives, resulting in both a changed body and a changed lifeworld, through their bodies’ relation to their respective worlds, an interpretation that can be made based on Merleau-Ponty’s (2012) theories (see also Toombs, 1992). For each of them, the world has become smaller, and this has had a significant impact on how they perform their various activities and carry out their role in society.

For all of them, either a key incident or a specific need or wish underpinned their determination to master O&M skills. For example, Alice had got lost in a hotel during a power failure but was also very determined to be mentally and practically prepared for the future and skilled in O&M techniques as her vision continued to deteriorate. Dick once fell off a bench and injured his knee, where he realized his shortcomings to walk independently. Peter had got tired of lounging around the house and being dependent on others for walking outdoors, so had requested a guide dog. This is why he should get to know the neighborhood and learn how to find his way around. To sum up, each of them had very strong motives and needs for learning long cane skills.

### Starting to Use the Long Cane: The Traditional Symbol of Blindness

When someone starts learning to use a long cane, this is often associated with feelings about the cane being white. The encounter with the cane is thus not primarily an encounter with a physical tool but includes also the encounter with a cane that, through its white color, contains a historical and cultural implication of representing the fact that its user is blind and the associated notions of blindness.

Alice: So, it was probably … but I was probably, I must say, I was probably a bit like that, I thought should I start now with this white cane, that’s what you had … you know, that this is a blind cane or this is something like this …. So that it’s … but I've become more and more used to it during the time I’ve been here, so it’s become more and more natural for me.

At first, the long cane exacerbated feelings of inferiority. Alice felt that she is not as valuable as a human being now that she has a visual impairment. The white cane thus contributes to a change in how she sees herself precisely through what she feels it conveys.

Alice: Well, it [the cane] probably gives me confirmation of the original feeling, that’s what I was going to say I was going to get to and that you … when you have a disability, you’re not a whole person. You’re not a whole … I don’t think I’m a whole … I don’t want to say adequate or complete person because I have these disabilities. And that’s what it feels like, that’s what gives you this nagging feeling that somehow you don’t have the same human dignity, you’ve become a little less, a little lower, a little … well, you’re not a whole, complete person.

After a while, she became more and more familiar with it, but that initial feeling that she was a disabled person, rather than a whole human being, was for a long time reinforced by the long cane. To her, it was proof of her inferiority as a disabled person and citizen. This feeling was exaggerated further when she, on her own initiative, wore a blindfold for the first time. In her diary, she describes this experience as follows:

Alice: Well, when I first put on the blindfold and felt total darkness around me, that was when I really felt my personality was going through a kind of transformation or, how can I express it … suddenly I became a much smaller human being, surrounded by a vast, dark, unknown vacuum. I felt, well, totally diminished. All my self-confidence was pouring out of me.

I had also noticed that Alice stopped and stiffened in the body during my observation of the O&M training and was eager to talk with her about it. This experience made Alice relive the feeling that she was dependent on others. It also shows that we as human beings experience ourselves in relation to the milieu around as, as a lived body in a lived room. She said, “I can’t really describe it, but I had a feeling of shrinking, of being in some way smaller,” a comment which reveals how we experience the world through our bodies.

The key problem in a visually impaired person’s new world is often the way in which they feel they are now de-valued as people. Peter expressed the feeling that because the vast majority of people in society are sighted, those who are visually impaired or already blind are looked upon as second-class citizens, who have less worth than those who are sighted. This highlights the social dimensions of disability.

Peter also felt ashamed of his long cane when he first got it, some years before the O&M training took place. He did not use the cane for several years.

Peter: I was very ashamed of the cane. I didn’t dare show it. I didn’t want to show that I was visually impaired and people looked at me. People do that too, they look at you when you have a cane and you don’t want to be seen like that, you don’t want to do that, but if I walk, I don’t want people to look at me. And I felt that people were looking at me all the time, it felt like that. It was probably … you … I went without a cane for far too long.

Peter talks about two worlds, the sighted world as he used to belong to and the blind world, which he now is approaching with the help of his long cane. He also emphasizes, in the same way as Alice, that the blind world is somehow of less value than the sighted world, and people with visual impairment are valued less accordingly. He says it’s about forgetting the old world, but the new world can never be 100% because society is so focused on visual perception. His feeling about this is: “Yes, it’s sad, really sad.”

These two examples indicate how the cane is a social symbol of disablement and blindness, something also emphasized by Berndtsson (2018), Bäckman (2023), and Kudlick (2005). However, for Dick it was different: he had none of these negative feelings about the color of the cane. Instead, he was overjoyed when he learned about how the long cane could function for him, something he had really needed since his teens.

Except the sometimes strenuous experience of disablement, this initial often ambivalent phase of learning to use a long cane is also embedded with practical training sessions where the participants shall learn how to use the cane. Dick’s training took place in a special room where he learned how to move the cane in front of him. He continues in long corridors where he learns to walk straight and in rhythm. He struggles to keep his feet straight. Before he had the long cane, he was used to feeling the ground in front of him with his feet: “I’ll have to strain my legs,” he says. To get a more authentic training situation, he uses dark glasses.

### Using the Long Cane Promotes Adaptation to Visual Impairment

The long cane is a very important tool in the process of adapting to vision loss. This could also be interpreted as being on your way to inhabit a new body and world. For Alice, repeated use of the long cane in public helped her to adapt to her functional limitations. Using the cane has taught her to view the world in a new way, being more observant for details. But using the cane has also helped her to learn about herself and promote acceptance of both her visual impairment and hearing impairment. Once she told the instructor during an O&M lesson, “What I’m learning here has made me accept my hearing loss. I didn’t want to admit it. What a shame. It’s just as well to tell it like it is.” This can be described as a co-function of using the long cane. As she learned how to use her cane, she simultaneously recovered her dignity as a human being. She began to notice that her attitude toward the long cane had gradually changed. This meant she was coming to terms with her visual impairment: “A big step forward is letting everyone see your visual impairment in public,” she says. In making the decision to use the long cane, Alice had finally accepted her impaired vision and could now go on with her life. The long cane was the catalyst that convinced her to go on living. Besides being a mobility device, it also helped her to adapt to her new life. Now she talks about herself as a new and more nuanced person, as a whole person but in a different way than before. This shows that, from the visually impaired person’s point of view, the long cane can be the lifeline she or he needs, to give them the courage to start their new life.

Peter expressed something similar. Once he had learned how to use the long cane, he suddenly felt naked without it. But it is also obvious that the learning process includes approaching your new situation as a visually impaired person.

Peter: No, forgetting is not the same either, maybe … or you’re afraid of … you’re afraid of the other side. You don’t really want to go in there. No one wants that, they don't want to become blind. But if you are there and you still have to go into that side. You become less and less afraid, so you let go less and less on one side. It is well that you might taste a little bit on the other side, so it was not so bad anyway then. So, you let go mentally on the other side too. Yes.

The only thing that held Peter back from using the cane, he says, was his reluctance to show in public that he was handicapped. For Dick, using the long cane helped him to recover his self-esteem. By using it in public, he was accepting his visual impairment.

Dick: Yes, no, I think it has become a bit because of it as well … as I feel, you feel more safety. The more you use it, the more security you get. And then I think you accept that you see badly.

All of this shows how existential and intersubjective or social dimensions are intertwined when a visually impaired individual uses a long cane in public.

Because of the emotional and existential aspects of blindness, the participants suggested that they should be able to talk about their O&M training session with a psychologist or similar professional. Peter’s lay statement, “The body won’t walk outdoors if the mind is not ready for it,” is an example of how an integrated lived body is a necessity for the performance of an activity. It also suggests that the research interviews may have played an important role in encouraging participants to reflect regularly on their experience, which was also mentioned by the participants, and highlights the importance of considering the relationship between body and mind in interpreting how new skills are learned. The cane here becomes a tool to become a subject again. Using the long cane in a group together with next of kins also contributed to enhanced adaptation. All of the training sessions observed took place in an atmosphere of laughter, fun, and openness, which was crucial to the outcome.

### A New Habitual Body

At the start of the O&M training, the focus was primarily on learning motor skills. Alice says, “The long cane was not my pal.” During her O&M training, I observed a key event where she almost fell on the floor. With the help of the cane, Alice had been led to believe that there was some kind of elevation in a corner of the corridor, but this turned out not to be true. About that incident, she afterward told me:

Alice: So reality is so different from what you experience it to be, and that’s perhaps what makes you small as a person too, because you know that I can’t see this, I can’t feel it, I can’t experience it, I can’t form a correct idea of what it looks like, and then it becomes frightening, you become afraid, you become insecure.

That incident made her observant on that she also needs to use her hands when travelling in unknown environments and not only the cane. It is obvious that the perceptual part of using a long cane only develops once the motor functions have been established. As Alice expressed it, “That it gives me information by itself by being there if I use it in the right way, it has started to come now. /…/ That I take in what information it provides, more this with sound and feeling.” After a while, she gradually experienced two-way communication with her cane.

Obviously, learners must concentrate on a number of learning tasks separately; for example, when learning the two-point-touch technique, it is hard to combine this with perceptual learning. However, once someone has been trained, the long cane responds more and more automatically until it becomes a habit: “I feel as if it’s become a part of me now—it’s more natural,” says Alice. This utterance of lived experience is very much in line with Merleau-Ponty’s (2012) description: “The stick is no longer an object perceived by the blind man, but an instrument *with* which he perceives” (p. 152). This frees the visually impaired person to concentrate on his or her surroundings by means of the other senses.

Peter describes his experience in similar terms. He is more relaxed now and has even started to make use of echolocation: “I can hear lamp-posts and things like that,” he says. It is only once using the long cane has become a habit that the visually impaired or blind person is free to pay attention to the environment by using the other senses to the full: “One can feel the ground, one feels that this is grass, and quite suddenly one can see … actually see the grass.” Now, Peter describes the cane as a part of himself: “The stick is now almost a part of Peter, an extra limb.” This awareness of his other senses has also made him more curious about his surroundings, building on the two-way communication with the world via the cane. Step by step also Dick learns how to use the long cane.

Dick: Ah, you have it … feel it more forward than you just hold something in front of you. Because then you know if you hold something in front of you, it’s just … that you don’t step on anything. But here you can feel that here is gravel and there is asphalt and there is grass and all this … because it sweeps much bigger than just holding something in front of you.

Dick tells me that the process of learning to use a long cane involves both mental and motor activity: “It has to settle in your head and legs,” he tells the instructor. The use of the cane also makes him aware of the extent of his visual impairment, something he had not previously realized. For him, this deals with learning a new habit, to relearn how to walk and how to use his remaining vision: “Yes, I’ve got a tool and skills that I can see with. Then you stop using your feet, so to speak.”

Dick’s trust in his long cane sneaked up on him, but he says that once he trusted it, it opened up a host of new possibilities. As he lacks twilight vision, Dick’s learning first involved learning *not* to use his feet to feel the ground under his feet, as he had done for many years: “The senses used to be here,” he says and points to his feet. He had to relearn established motor habits such as walking and orienting himself by dragging and scuffing his feet. He now knows where he is because he is able to use his long cane to feel the ground under his feet rather than feeling it with his feet. He has also relearned to direct his gaze more constructively and functionally to solve orientation difficulties. This is very much in line with Merleau-Ponty’s (2012) assertion that “in the gaze we have at our disposal a natural instrument analogous to the blind man’s stick” (p. 153). Dick kept returning to his need for this new knowledge to be lodged in his brain and in his spine: “The more one practices, the better one gets at it, right?” he says. This can be interpreted as developing new habitual routines. Learning to use a long cane can thus be seen as a process of developing a new habitual body which enables people to get started in their new and changed world. The habit is thus embedded in the body as a mediator of a world.

### Inhabiting the New World Through Activity

In the long run, learning and practicing O&M skills can be interpreted as building and expanding a new world through the individual’s own perception and above all activity. Habitual use of the long cane can therefore be the key that gives access to and conquers this new world in an ongoing way. Peter says, “You begin to sever your connections with the old world and eventually you believe the cane is your eyes.” He compares the cane to the ant’s antennae. This is where the two-way communication between the individual and the world around him, via the cane, is crucial. Peter even states that his O&M training has opened up a new world to him. Walking around helps Peter build his new world: “That makes the world real,” he says, and compares the use of the long cane to vision. His feeling is as getting out of jail or solitary confinement: “Now I feel as if I’m free of my leg-iron, that’s how it feels,” he says. This is about embracing a new world through movement and activity. It is through the incorporation of the cane into the body and the gradual expansion of the outer horizon that it is possible to expand the previously limited world and its often limited horizons. The cane thus becomes the tool that helps to widen the horizon and the world. Perceptually, the long cane will be a tool for experiencing and widening that world, and one that makes it possible for Peter to step into and inhabit the blind world. Of course, the world has always been out there, but when they make the world their world––their own lifeworld––the person starts being part of it: “That’s what motivates me, that my new reality becomes a world,” says Peter. It is primarily through activity that the new world is conquered. Becoming active again obliges the individual to renew his or her relationship with the surrounding world and thus abandon their often passive state.

Peter: It’s a huge thing. If you can’t go outside, you can’t smell the trees, you can’t feel the grass, the tarmac, you can’t stub your toe on an edge or step in a puddle, feel the dog shit, whatever.

For Alice, thanks to the long cane, there are openings to a different approach.

Alice: … perceive myself as a whole person but in a different way /…/ but I have learnt to somehow open my eyes and see, there are so many other things.

Overall, you could say that learning to use a long cane is about inhabiting the world anew and make it your own world. It is about actively entering the new world—discovering the world with one’s own new body, through movement, perception, and activity. This form of activity could be compared to what is described and expressed by Schutz (1962): “By my working acts I gear into the outer world, I change it” (p. 227). In this process, the long cane can come to be seen as a tool that contributes to a new social identity, re-enabling the participants to perform daily activities. Now there is no longer the fear of showing off with the white cane. Alice says, “Yes, but that doesn’t bother me at all, not one bit.” The ambivalence toward the white cane is no longer prominent. Inhabiting and acting in the new world also includes a natural handling of the cane.

Dick: Instead, you feel … you have all that confidence in front of you, or in your fist if you like, when you walk and sweep it [the long cane] in front of you. Then you just need to focus on looking straight ahead.

## Discussion

In this study, the picture that emerges of the process of learning to use the long cane differs from more traditional ways of describing this process, for example, as presented by Jacobson (2013), Sauerburger and Bourquin (2010), and Wiener et al. (2010). In these texts, human behavior and cognition is generally the starting point for theorizing, implying a largely technical approach to O&M training (Hersh, 2015). In this study, quite a different picture has emerged. First of all, it seems that learning to use the long cane must be related to the individuals’ lifeworld and how they have experienced their visual impairment or blindness. From this perspective, learning is unlikely to be similar for all individuals, in contrast to the process of simply technically acquiring a new skill. One consideration that this research has identified is that any trauma associated with becoming visually impaired or blind must be faced before the individual is ready to learn to use the long cane. This highlights the integration of mind and body. The learning process involves coming to terms with changed body–world relationships (Bengtsson & Berndtsson, 2015) and then adopting the long cane as a tool for discovering, perceiving, and building these and conquering the new world, as earlier pointed out by Berndtsson (2018). This is a very different view of the long cane than simply seeing it as a technical instrument. The physically embodied character of the long cane becomes incorporated into the person’s own body, in accordance with Merleau-Ponty’s theory (2012). The research has also highlighted the close connection between human existence and the sociality of the lifeworld (compare Schutz, 1962), which becomes particularly apparent when someone starts using the long cane in public. This makes their visual impairment visible to others, and impossible to hide, which has previously been recognized by Bäckman (2023). At the same time, mastery of the cane promotes the person’s adaptation to their visual impairment.

In terms of learning, this study has shown that various dimensions of the lifeworld are involved: perceptual, bodily, existential, and social. These dimensions are identified and intertwined in various ways in participants’ accounts of their experience of learning O&M skills. However, the training mainly paid attention to bodily or motor activity and perception: psychosocial, or existential and social, aspects were not explicitly dealt with.

In the Results section, lifeworld theory was used to anchor and underpin participants’ experiences within a theoretical context (Bengtsson, 2013b). Throughout the study, it has been possible to interpret lived experience through the lens of lifeworld phenomenological concepts and theory because of the resonances between the participants’ narratives and those ideas (Berndtsson & Vikner Stafberg, 2022). A key insight is that learning the long cane technique is a learning activity which brings the individuals and the changed world closer together, making it more possible for those individuals to function within that new world.

It is important to note that this is just a first attempt to interpret the process of learning O&M skills using lifeworld phenomenology; further research is needed to develop and challenge the interpretations presented here. However, the results are strengthened by their coherence with findings from other studies which consider how assistive technology such as wheelchairs is integrated into the human body (Papadimitriou, 2018; Standal, 2011).

## Conclusion

As highlighted earlier, the long cane is a tool that contributes much to an integrated body–world relationship and a healthier life and becomes a lifeline into a new life. This insight should be taken seriously within rehabilitation initiatives working in these fields. It also suggests there is a need for change in the prevalent view of O&M learning as a technical or a physical task. Learning to use a long cane deals with the whole lived body in the world, as well as individual’s life-stories and ability to adapt. This implies that rehabilitation initiatives should include reflective moments, such as those this study has been able to offer to the research participants. It is necessary to listen to the narratives of the learning individual and from that perspective decide what will be the best training outcome for each person’s life situation. This will help individuals to integrate or intertwine mind and body when learning O&M skills. Using a long cane in a skillful way could also contribute to a situation in which the visually impaired or blind person becomes respected as a competent and equal person, a process that largely relates to changing attitudes among other members of society (Berndtsson, 2016; dos Santos et al., 2022; Schutz, 1967).

In conclusion, this study may be a contribution toward addressing what Welsh (2010) has identified as a knowledge shortage within O&M, namely, the psychosocial dimension, in that it has shown that psychological adaptation, existence, and identity are not phenomena that can be isolated from the process of O&M learning. Instead, the results presented here have given insights into the intertwined and complicated pedagogical processes when learning to use a long cane, processes which include dimensions of existence, feelings, perception, activity, and sociality. These aspects have not previously been described as intertwined in the literature.

## Notes

1. This study is a separate part of a larger piece of research, the purpose of which is to study and elucidate pedagogical processes that take place during rehabilitation.

2. Six persons participated in the empirical study within the wider research project, but they are not all included in this presentation as they did not participate in long cane O&M training.

## References

[bibr1-10497323241277111] Bäckman M. (2020). Vita käppen – Redskapet som hjälper och stjälper: Om kroppar, skam, normalitet och tingens agens [The white cane – A tool that both helps and hinders. Bodies, shame, normality and the agency of objects]. Budkavlen, 98(1), 39–64. 10.37447/bk.98443

[bibr2-10497323241277111] Bäckman M. (2023). Living with an iconic aid: The case of the white cane. Culture Unbound, 15(2), 88–114. 10.3384/cu.4233

[bibr3-10497323241277111] Bäckman M. (2024). The white cane: An ethnographic account on the widespread ambivalence amongst visually impaired towards an iconic aid. Scandinavian Journal of Disability Research, 26(1), 82–94. 10.16993/sjdr.1024

[bibr4-10497323241277111] Beck M. Martinsen B. Missel M. Simony C. Engelke E. van Manen M. (2024). Alongside: Exploring the meaningfulness of significant moments in others’ lives through observation and interview. Qualitative Health Research, 34(8–9), 707–716. 10.1177/10497323231210495 38130185

[bibr5-10497323241277111] Beekman T. (1984). Hand in Hand mit Sasha; über Glühwürmchen, Grandma Millie und einige andere Raumgeschichten. Im Anhang; uber teilnehmende Erfahrung [Hand in hand with Sasha: About fireflies, Grandma Millie and some other room faces. In appendix; about participant experience]. In Lippitz W. Meyer-Drawe K. (Eds.), Kind und Welt. Phänomenologische Studien zur Pädagogik (pp. 11–25). Scriptor.

[bibr6-10497323241277111] Bengtsson J. (2005). En livsvärldsansats för pedagogisk forskning [A lifeworld approach for research in education]. In Bengtsson J. (Ed.), Med livsvärlden som grund [With the lifeworld as ground] (pp. 9–58). Studentlitteratur.

[bibr7-10497323241277111] Bengtsson J. (2013a). Embodied experience in educational practice and research. Studies in Philosophy and Education, 32(1), 39–53. 10.1007/s11217-012-9328-1

[bibr8-10497323241277111] Bengtsson J. (2013b). With the lifeworld as ground. A research approach for empirical research in education: The Gothenburg tradition. Indo-Pacific Journal of Phenomenology, 13(sup1), 1–18. 10.2989/IPJP.2013.13.2.4.1178

[bibr9-10497323241277111] Bengtsson J. Berndtsson I. C. (2015). Elevers och lärares lärande i skolan – Livsvärldsliga grunder [Students and teachers learning in school – Lifeworld phenomenological basis]. In Bengtsson J. Berndtsson I. C. (Eds.), Lärande ur ett livsvärldsperspektiv [Learning from a lifeworld perspective] (pp. 15–34). Gleerups.

[bibr10-10497323241277111] Berndtsson I. (2001). Förskjutna horisonter. Livsförändring och lärande i samband med synnedsättning eller blindhet [Displayed horizons. Life changes and learning related to visual impairment or blindness] (Gothenburg Studies in Educational Sciences, 159) [Doctoral dissertation, University of Gothenburg]. Acta Universitatis Gothoburgensis. http://hdl.handle.net/2077/15271

[bibr11-10497323241277111] Berndtsson I. C. (2016). Researching equity and inequity: The voices of marginalised people. In Beach D. Dyson A. (Eds.), Equity & education in cold climates (pp. 80–94). The Tufnell Press.

[bibr12-10497323241277111] Berndtsson I. C. (2018). Considering the concepts of the lived body and the lifeworld as tools for better understanding the meaning of assistive technology in everyday life. ALTER, 12(3), 140–152. 10.1016/j.alter.2018.01.001

[bibr13-10497323241277111] Berndtsson I. C. Claesson S. Friberg F. Öhlén J. (2007). Issues about thinking phenomenologically while doing phenomenology. Journal of Phenomenological Psychology, 38(2), 256–277. 10.1163/156916207X234293

[bibr14-10497323241277111] Berndtsson I. C. Vikner Stafberg M. (2022). Livsvärldsfenomenologisk belysning av abduktion inom pedagogisk forskning: Exemplet lärarblivande [Lifeworld phenomenological reflection of abduction in educational research. The example of becoming a teacher]. Pedagogisk forskning i Sverige, 27(4), 62–82. 10.15626/pfs27.04.04

[bibr15-10497323241277111] Bouman J. C. (1962). Psykologiska synpunkter på den blinda människans värld [Psychological aspects on blind people’s world]. De Blindas Tidskrift (1), 1–12.

[bibr16-10497323241277111] Brunes A. Falkenberg H. K. Berndtsson I. C. Heir T. (2024). Use and underuse of mobility aids in individuals with visual impairment: A cross-sectional study of a Norwegian sample. Disability and Rehabilitation: Assistive Technology, 19(2), 266–272. 10.1080/17483107.2022.2081735 35713634

[bibr17-10497323241277111] dos Santos A. D. P. Ferrari A. L. M. Medola F. O. Sandnes F. E. (2022). Aesthetics and the perceived stigma of assistive technology for visual impairment. Disability and Rehabilitation: Assistive Technology, 17(2), 152–158. 10.1080/17483107.2020.1768308 32501732

[bibr18-10497323241277111] Eastmond M. (1996). *Luchar y Sufrir* – Stories of life and exile: Reflexions on the ethnographic process. Ethnos, 61(3–4), 231–250. 10.1080/00141844.1996.9981537

[bibr19-10497323241277111] Finlay L. (2009). Debating phenomenological research methods. Phenomenology & Practice, 3(1), 6–25. 10.29173/pandpr19818

[bibr20-10497323241277111] Fourie R. J. (2007). A qualitative self-study of retinitis pigmentosa. British Journal of Visual Impairment, 25(3), 217–232. 10.1177/0264619607079794

[bibr21-10497323241277111] Friberg F. Öhlén J. (2010). Reflective exploration of Beekman’s participant experience. Qualitative Health Research, 20(2), 273–280. 10.1177/1049732309354988 20065310

[bibr22-10497323241277111] Goffman E. (1972). Stigma. Den avvikandes roll och identitet [Stigma. Notes on the management of spoiled identity]. Rabén & Sjögren.

[bibr23-10497323241277111] Hammer G. (2016). “If they’re going to stare, at least I’ll give them a good reason to”: Blind women’s visibility, invisibility, and encounters with the gaze. Signs, 41(2), 409–432. 10.1086/682924

[bibr24-10497323241277111] Hammersley M. Atkinson P. (2019). Ethnography: Principles in practice (4th ed.). Routledge.

[bibr25-10497323241277111] Heidegger M. (2013). Varat och tiden [Being and time] ( Jakobsson J. , Trans.). Daidalos. (Original published 1927).

[bibr26-10497323241277111] Hersh M. (2015). Cane use and late onset visual impairment. Technology and Disability, 27(3), 103–116. 10.3233/TAD-150432

[bibr27-10497323241277111] Husserl E. (1973). Experience and judgment. Northwestern University Press. (Original published 1948).

[bibr28-10497323241277111] Jacobson W. H. (2013). The art and science of teaching orientation and mobility to persons with visual impairments (2nd ed.). AFB Press.

[bibr29-10497323241277111] Jacobson W. H. Bradley R. H. (2010). Learning theories and teaching methodologies for orientation and mobility. In Wiener W. R. Welsh R. L. Blasch B. B. (Eds.), Foundations of orientation and mobility (3rd ed., Vol. 1, pp. 211–237). AFB Press.

[bibr30-10497323241277111] Karlsson G. (1999). Leva som blind [Live with blindness]. Carlssons.

[bibr31-10497323241277111] Kudlick C. (2005). The blind man’s harley: White canes and gender identity in America. Signs, 30(2), 1589–1606. 10.1086/423351

[bibr32-10497323241277111] Kvale S. Brinkmann S. (2009). Den kvalitativa forskningsintervjun [The qualitative research interview] (2nd ed.). Studentlitteratur.

[bibr33-10497323241277111] Merleau-Ponty M. (2012). Phenomenology of perception ( Landes D. A. , Trans.). Routledge. (Original published 1945).

[bibr34-10497323241277111] Mishler E. G. (1991). Research interviewing: Context and narrative. Harvard University Press.

[bibr35-10497323241277111] Ödman P.-J. (2007). Tolkning, förståelse, vetande. Hermeneutik i teori och praktik [Interpretation, understanding, knowledge. Hermeneutics in theory and practice]. Norstedts.

[bibr36-10497323241277111] Papadimitriou C. (2008). Becoming en-wheeled: The situated accomplishment of re-embodiment as a wheelchair user after spinal cord injury. Disability & Society, 23(7), 691–704. 10.1080/09687590802469420

[bibr37-10497323241277111] Parker A. T. Swobodzinski M. Wright J. D. Hansen K. Morton B. Schaller E. (2021). Wayfinding tools for people with visual impairments in real-world settings: A literature review of recent studies. Frontiers in Education, 6, Article 723816. 10.3389/feduc.2021.723816

[bibr38-10497323241277111] Penrod W. M. Burgin X. D. Wiener W. R. Siffermann E. Blasch B. (2023). Orientation and mobility competency agreements from 1983 to 2019: A comparative analysis of professional standards. Journal of Visual Impariment & Blindness, 117(4), 270–277. 10.1177/0145482X231188698

[bibr39-10497323241277111] Pettersson I. Berndtsson I. Appelros P. Ahlström G. (2005). Lifeworld perspectives on assistive devices: Lived experiences of spouses of persons with stroke. Scandinavian Journal of Occupational Therapy, 12(4), 159–169. 10.1080/11038120510031789 16457089

[bibr40-10497323241277111] Sauerburger D. Bourquin E. (2010). Teaching the use of a long cane step by step: Suggestions for progressive, methodical instruction. Journal of Visual Impairment & Blindness, 104(4), 203–214. 10.1177/0145482X1010400404

[bibr41-10497323241277111] Schutz A. (1962). The problem of social reality (Collected Papers I). Martinus Nijhoff.

[bibr42-10497323241277111] Schutz A. (1967). The phenomenology of the social world. Northwestern University Press. (Original published 1932).

[bibr43-10497323241277111] Standal Ø. F. (2011). Re-embodiment: Incorporation through embodied learning of wheelchair skills. Medicine, Healthcare & Philosophy, 14(2), 177–184. 10.1007/s11019-010-9286-8 20865328

[bibr44-10497323241277111] Taylor S. J. Bogdan R. DeVault M. L. (2016). Introduction to qualitative research methods: A guidebook and resource (4th ed.). Wiley.

[bibr45-10497323241277111] Toombs S. K. (1992). The meaning of illness. A phenomenological account of the different perspectives of physician and patient. Kluwer.

[bibr46-10497323241277111] van Manen M. (1997). Researching lived experience: Human science for an action sensitive pedagogy (2nd ed.). Althouse Press.

[bibr47-10497323241277111] van Peursen C. A. (1977). The horizon. In Elliston F. A. Mc Cormick P. (Eds.), Husserl: Expositions and appraisals (pp. 182–201). University of Notre Dame Press.

[bibr48-10497323241277111] Wainapel S. F. (1989). Attitudes of visually impaired persons toward cane use. Journal of Visual Impairment & Blindness, 83(9), 446–448. 10.1177/0145482X8908300906

[bibr49-10497323241277111] Welsh R. L. (2010). Psychosocial dimensions of orientation and mobility. In Wiener W. R. Welsh R. L. Blasch B. B. (Eds.), Foundations of orientation and mobility (3rd ed., Vol. I, pp. 173–201). AFB Press.

[bibr50-10497323241277111] Wiener W. R. Welsh R. L. Blasch B. B. (2010). Introduction. In Wiener W. R. Welsh R. L. Blasch B. B. (Eds.), Foundations of orientation and mobility (3rd ed., Vol. I, pp. xv–xx). AFB Press.

[bibr51-10497323241277111] Worth N. (2013). Visual impairment in the city: Young people’s social strategies for independent mobility. Urban Studies, 50(3), 574–586. 10.1177/0042098012468898

